# KLK2 single-nucleotide polymorphism rs198977 is associated with increased susceptibility and hyperleukocytosis in AML

**DOI:** 10.3389/fgene.2023.1218523

**Published:** 2023-08-01

**Authors:** Guangqiang Meng, Peng Li, Mingying Li, Yuyan Wu, Yuechan Ma, Tao Sun, Chunyan Ji

**Affiliations:** ^1^ Department of Hematology, Qilu Hospital, Shandong University, Jinan, China; ^2^ Shandong Key Laboratory of Immunohematology, Qilu Hospital, Shandong University, Jinan, China

**Keywords:** acute myeloid leukemia, kallikrein 2, single nucleotide polymorphism, hyperleukocytosis, susceptibility

## Abstract

**Introduction:** Acute myeloid leukemia (AML) is a heterogeneous myeloid malignancy with abnormal molecular diversity. Tissue kallikrein 2 (KLK2) is a kind of serine protease, and has a close relationship with the occurrence and development of malignant tumors. Single nucleotide polymorphism (SNP) of various genes are associated with susceptibility, treatment and survival of AML.

**Methods:** We investigated the association of KLK2 SNPs rs198977 and rs2664155 with AML. We recruited 284 AML patients and 280 healthy controls from the Han population and genotyping KLK2 SNPs rs198977 and rs2664155 by MassARRAY system.

**Results:** Using clinical data from AML patients and controls, including AML susceptibility, blood count, risk stratification, response to induced chemotherapy and survival, our results showed an increased risk of AML susceptibility with KLK2 rs198977 TT genotype in the recessive model. Regarding white blood cell counts in AML patients, the results showed an increased risk of hyperleukocytosis with the TT genotype of KLK2 rs198977 in a codominant model. Moreover, in the recessive model, AML with KLK2 SNPs rs198977 TT genotype had an increased risk of hyperleukocytosis. No significant correlation was found between KLK2 rs2664155 and AML.

**Discussion:** This study suggests that KLK2 rs198977 may be an important genetic factor in the occurrence of AML and hyperleukocytosis in AML, providing a new perspective for disease progression and new therapeutic targets.

## Introduction

Acute myeloid leukemia (AML) is a heterogeneous malignant tumor originating from hematopoietic stem cells, characterized by clonal proliferation, arrest of apoptosis and failure to differentiate of tumor cells (Newell and Cook, 2021). The diversity of abnormalities in molecular biology is one of the hallmarks of disease heterogeneity (Kayser and Levis, 2019). The classic 3 + 7 based combination chemotherapy regimen remains the first-line treatment option for AML (non-M3) (De Kouchkovsky and Abdul-Hay, 2016; Newell and Cook, 2021). In recent years, targeted drugs have been widely used in the treatment of AML, and have achieved good efficacy (Kayser and Levis, 2018). However, the survival of AML remains poor, with a 5-year survival rate of 30%–40% (Pulte et al., 2010). Therefore, it is of great significance to explore the new molecular biological and pathological mechanisms and to find new potential therapeutic targets.

Tissue kallikrein 2 (KLK2) is a kind of serine protease, which exists in various tissues and biological fluids. KLK2 has a variety of physiological functions, involved in physiological adaptation, physiological metabolism and regulation of brain development, and has a close relationship with the occurrence of a variety of malignant tumors (Diamandis et al., 2004; Avgeris et al., 2012; Adamopoulos et al., 2019). Recent studies have shown that KLK2 is involved in the development and metastasis of prostate cancer and is associated with the prognosis of patients (Bonk et al., 2020; Boyukozer et al., 2020). Moreover, the increased expression of KLK2 in tumor tissues is associated with the increased proliferation rate and decreased apoptosis index of prostate cancer cells (Shang et al., 2014). Recent studies have found a single nucleotide polymorphism (SNP) in the KLK2 gene with an increased risk of prostate cancer (Kohli et al., 2010). SNPs are common genetic variants that play an important role in human diseases, especially in the development of malignant tumors (Zhang et al., 2022). Many studies have confirmed that SNPs of various genes are associated with susceptibility, prognosis and survival of AML (Wang et al., 2017; Liu et al., 2020). However, whether SNPs in KLK2 are associated with disease occurrence, progression, treatment, and prognosis of AML remains unclear.

To further explore the potential unique etiology of AML, we investigated the association of two important SNPs sites of KLK2 with AML, in 284 AML cases and 280 healthy controls. We analyzed the association between SNPs and disease susceptibility, blood routine, risk stratification, response to induction chemotherapy and survival of AML. Our study seeks to provide new insights into the molecular biological heterogeneity of AML patients and new potential therapeutic targets.

## Materials and methods

### Study participants

In this study, 284 AML patients (non M3) with a median age of 49 years (13–87 years) were recruited from the Department of Hematology, Qilu Hospital of Shandong University from May 2011 to December 2021. The diagnosis of AML patients was confirmed by the National Comprehensive Cancer Network (NCCN) guidelines (version 3.2017) (O'Donnell et al., 2017). Hyperleukocytosis was defined as a peripheral white blood cell count greater than 100 × 10^9^/L in AML patients. We used the 2017 European Leukemia Network (ELN) criteria to assess the assessment of post-chemotherapy response in AML (Döhner et al., 2017). And 280 healthy controls with a median age of 41 years (20–88 years) were enrolled in the study. The relevant characteristics of AML patients and healthy controls are shown in Table 1. This study was approved by the Medical Ethics Committee of Qilu Hospital, Shandong University. And written informed consent was obtained from the participants or their legal guardians.

**TABLE 1:** Demographic and clinical characteristics.

Variable	AML patients	Control
	n = 284	n = 280
Age (years, median, range)	49 (13–87)	41 (20–88)
Sex (M/F)	161/123	118/162
WBC (×10^9^/L)	18.6 (1.0–452.0)	N/A
HGB (g/L)	77.0 (21.0–148.0)	N/A
PLT (×10^9^/L)	38.5 (2.0–235.0)	N/A
Cytogenetics and molecular stratification		
Low risk (*n*, %)	60 (21.1)	N/A
Intermediate risk (*n*, %)	147 (51.8)	N/A
High risk (*n*, %)	77 (27.1)	N/A
Response after the first course of induction therapy		
CR (*n*, %)	108 (56.5)	N/A
No CR (*n*, %)	83 (43.5)	N/A

AML, acute myeloid leukemia; WBC, white blood cell; PLT, platelet; HGB, hemoglobin; CR, complete remission; N/A, not applicable.

### DNA extraction and genotyping

The genomic DNA in the study was isolated from bone marrow mononuclear cells (BMMCs) by the TIANamp Blood DNA Kit, following the manufacturer’s instructions during the procedure (Tiangen Biotechnology, China). We measured the extracted DNA concentration with a spectrophotometer (DeNovix, United States). SNP genotyping was analyzed using Sequenom iPLEX and ffight (MALDI-TOF) MassARRAY platform based on matrix-assisted laser desorption/ionization time (BGI Technology, China). The platform is based on multiplex polymerase chain reaction (PCR) reactions, site-specific single base extension reactions, and MALDI-TOF spectroscopy. The primers of HK2 rs198977 and rs2664155 were designed as follows:

KLK2 rs198977 forward: ACG​TTG​GAT​GAA​AAG​CCT​GCT​GTG​TAC​ACC.

KLK2 rs198977 reverse: ACG​TTG​GAT​GTG​CGA​TGG​TGT​CCT​TGA​TCC.

KLK2 rs2664155 forward: ACG​TTG​GAT​GTG​GAG​AGA​GGT​CCT​TGA​AAG.

KLK2 rs2664155 reverse: ACG​TTG​GAT​GCC​AGC​TGC​TTT​ACT​AAA​GAG.

### Statistics

SPSS 20.0 software (SPSS Inc. Chicago, IL, United States) was used for statistical analyses. Hardy-Weinberg equilibrium (HWE) of KLK2 rs198977 and rs2664155 genotype in control group was determined using Pearson’s goodness-of-fit chi-square test. The 4 models (codominant, recessive, dominant, and allelic) were used to analyze genotyping data. We compared the distribution of KLK2 SNPs with AML sensitivity, white blood cell count, hemoglobin, platelet count, risk stratification, response to induction chemotherapy, and prognosis using chi-square test or Fisher exact test. The univariate binary logistic regression analysis was used to analyze odds ratios (ORs) with corresponding 95% confidence intervals (95%CI), adjusted for age and sex. The overall survival (OS) was estimated by Kaplan-Meier curves. Two-tailed *p* values <0.05 were defined as statistically significant.

## Results

### Study population

The clinical and demographic details of AML patients and controls are presented in Table 1. The median of white blood cell (WBC) in AML patients was 18.6 (1.0–452.0) × 10^9^/L, with 77.0 (21.0–148.0) g/L of the median hemoglobin and 38.5 (2.0–235.0) × 10^9^/L of the median platelet. Of all AML patients enrolled in this study, 60 cases were low-risk of cytogenetics and molecular stratification, 147 cases were intermediate-risk, and 77 cases were high-risk. Among AML patients who received induction chemotherapy, 108 achieved complete response (CR) after the initial course of induction chemotherapy. Two SNPs of KLK2 (rs198977 and rs2664155) were consistent with HWE of healthy controls (*p* > 0.05).

### Relationship between KLK2 gene polymorphism and AML susceptibility

We used 4 gene models to analyze the relationship between KLK2 SNPs (rs198977 and rs2664155) and AML susceptibility. First, we applied the chi-square test or Fisher exact test for screening, and the results show that recessive genotypic of KLK2 rs198977 was significantly associated with AML susceptibility (*p* = 0.039, Table 2). There was no significant difference in genotype and allele distribution of KLK2 rs2664155 (*p* = 0.594, *p* = 1.000, *p* = 0.499, *p* = 0.807, Table 2). And, univariate binary logistic regression analysis and adjusting for age and sex showed that TT genotypes in recessive models of KLK2 rs198977 showed an increased risk of AML susceptibility (OR 2.641; 95% CI 1.092–7.577; *p* = 0.031, Table 2).

**TABLE 2:** Association between KLK2 gene polymorphism and AML susceptibility.

Gene, SNP	Genotype, allele	AML group		Control group		Model	χ^2^ test *p*-value	OR (95% CI)	Adjusted *p*-value
		Count	%	Count	%				
KLK2, rs198977	CC	180	63.38	185	66.07	Co-dominant	0.128		
	TC	90	31.69	90	32.14	Dominant	0.504		
	TT	14	4.93	5	1.79	Recessive	0.039	2.641 (1.092–7.577)	0.031
	C	450	79.23	460		Allele	0.215		
	T	118	20.77	100					
rs2664155	GG	249	87.68	246	87.86	Co-dominant	0.594		
	AG	33	11.62	34	12.14	Dominant	1.000		
	AA	2	0.70	0	0	Recessive	0.499		
	G	531	93.49	526	93.93	Allele	0.807		
	A	37	6.51	34	6.07				

AML, acute myeloid leukemia; SNP, single nucleotide polymorphism; OR, odds ratio.

### Association between hyperleukocytosis and SNPs in AML

Hyperleukocytosis in AML is generally defined as white blood cell count greater than 100 × 10^9^/L in AML and is often associated with life-threatening complications such as leukostasis, tumor lysis syndrome (TLS) and disseminated intravascular coagulation (DIC). Patients with Hyperleukocytosis of AML often have poor prognosis. We analyzed the relationship between KLK2 SNPs and hyperleukocytosis. Among the recruited AML cases, 55 cases had hyperleukocytosis at diagnosis and 229 cases did not have hyperleukocytosis. After initial screening by chi-square test or Fisher exact test, we found that codominant and recessive of KLK2 rs198977 were related to hyperleukocytosis (*p* = 0.018 and 0.008, Table 3). KLK2 rs2664155 was not associated with hyperleukocytosis of AML (*p* = 1.000, *p* = 0.765, *p* = 1.000, *p* = 0.655, Table 3). After univariate binary logistic regression analysis and adjusting for age and sex, TT genotypes in both codominant and recessive models of KLK2 rs198977 were significantly associated with hyperleukocytosis in AML patients (*p* = 0.011 and 0.006, Table 3). And TT genotypes in codominant and recessive models of KLK2 rs198977 showed an increased the risk of hyperleukocytosis in AML patients (OR = 4.265, 95% CI = 1.402–12.969, *p* = 0.011; OR = 2.151, 95% CI = 1.245–3.715, *p* = 0.008; Table 3).

**TABLE 3:** Association between KLK2 gene polymorphism and WBC in AML.

Gene, SNP	Genotype, allele	WBC ≥ 100 × 10^9^/L		WBC < 100 × 10^9^/L		Model	χ^2^ test *p*-value	OR (95% CI)	Adjusted *p*-value
		Count	%	Count	%				
KLK2, rs198977	CC	34	61.82	146	63.76	Co-dominant	0.018	0.775 (0.392–1.532)	0.464
								4.265 (1.402–12.969)	0.011
	TC	14	25.45	76	33.19	Dominant	0.789		
	TT	7	12.73	7	3.06	Recessive	0.008	2.151 (1.245–3.715)	0.006
	C	82	74.55	368	80.35	Allele	0.178		
	T	28	25.45	90	19.65				
rs2664155	GG	49	89.09	200	87.34	Co-dominant	1.000		
	AG	6	10.91	27	11.79	Dominant	0.722		
	AA	0	0	2	0.87	Recessive	1.000		
	G	104	94.55	427	93.23	Allele	0.616		
	A	6	5.45	31	6.77				

AML, acute myeloid leukemia; WBC, white blood cell; SNP, single nucleotide polymorphism; OR, odds ratio.

### Association between hemoglobin, platelet and SNPs in AML

In this study, we considered HGB less than 60 g/L as low HGB and ≥60 g/L as high HGB. For the PLT count, lower than 20 × 10^9^/L was considered as low PLT, and the PLT count ≥20 × 10^9^/L was considered as intermediate or high PLT. We analyzed the relationship between KLK2 SNPs and HGB/PLT levels. After initial screening using the chi-square test or Fisher’s exact test, there was no significant difference in the frequency of KLK2 SNPs (rs198977 and rs2664155) between the high and low HGB groups (*p* > 0.05, Table 4). The frequency of KLK2 SNPs (rs198977 and rs2664155) was also not significantly different between the moderate or high PLT group and the low PLT group (*p* > 0.05, Table 4).

**TABLE 4:** Association between KLK2 gene polymorphism and HGB/PLT in AML.

Gene, SNP	Genotype, allele	HGB < 60 g/L		HGB ≥ 60 g/L		Model	χ^2^ test *p*-value
		Count	%	Count	%		
KLK2, rs198977	CC	23	57.5	157	64.34	Co-dominant	0.126
	TC	17	42.5	73	29.92	Dominant	0.405
	TT	0	0	14	5.74	Recessive	0.231
	C	63	78.75	387	79.3	Allele	0.910
	T	17	21.25	101	20.7		
rs2664155	GG	36	90	213	87.3	Co-dominant	1.000
	AG	4	10	29	11.89	Dominant	0.798
	AA	0	0	2	0.82	Recessive	1.000
	G	76	95	455	93.24	Allele	0.554
	A	4	5	33	6.76		

Gene, SNP	Genotype, Allele	PLT < 20 × 10^9^/L		PLT ≥ 20 × 10^9^/L		Model	χ^2^ test *p*-value
		Count	%	Count	%		
KLK2, rs198977	CC	30	56.6	150	64.94	Co-dominant	0.196
	TC	22	41.51	68	29.44	Dominant	0.256
	TT	1	1.89	13	5.63	Recessive	0.480
	C	82	77.36	368	79.65	Allele	0.599
	T	24	22.64	94	20.35		
rs2664155	GG	48	90.57	201	87.01	Co-dominant	0.876
	AG	5	9.43	28	12.12	Dominant	0.478
	AA	0	0	2	0.87	Recessive	1.000
	G	101	95.28	430	93.07	Allele	0.406
	A	5	4.72	32	6.93		

AML, acute myeloid leukemia; HGB, hemoglobin; PLT, platelet; SNP, single nucleotide polymorphism.

### Association between KLK2 gene polymorphism and risk stratification of AML

According to NCCN clinical practice guidelines, AML patients were divided into low-risk, intermediate-risk, and high-risk prognostic groups based on molecular and karyotypic abnormalities (O'Donnell et al., 2017). In this study, we analyzed the association between SNPs of KLK2 and risk stratification. We conducted a preliminary screening by chi-square test or Fisher exact test under the above four models. KLK2 rs198977 (*p* = 0.688, *p* = 0.846, *p* = 0.419, *p* = 0.863, Table 5) and rs2664155 (*p* = 0.822, *p* = 0.588, *p* = 1.000, *p* = 0.519, Table 5) had no significant differences in genotype and allele frequencies between the three groups.

**TABLE 5:** Association between KLK2 gene polymorphism and risk stratification of AML patients.

Gene, SNP	Genotype, allele	Favorable		Intermediate		Adverse		Model	χ^2^ test *p*-value
		Count	%	Count	%	Count	%		
KLK2, rs198977	CC	37	61.67	92	62.59	51	66.23	Co-dominant	0.688
	TC	19	31.67	50	34.01	21	27.27	Dominant	0.846
	TT	4	6.67	5	3.4	5	6.49	Recessive	0.419
	C	93	77.5	234	79.59	123	79.87	Allele	0.863
	T	27	22.5	60	20.41	31	20.13		
rs2664155	GG	55	91.67	127	86.39	67	87.01	Co-dominant	0.822
	AG	5	8.33	19	12.93	9	11.69	Dominant	0.588
	AA	0	0	1	0.68	1	1.3	Recessive	1.000
	G	115	95.83	273	92.86	143	92.86	Allele	0.519
	A	5	4.17	21	7.14	11	7.14		

AML, acute myeloid leukemia; SNP, single nucleotide polymorphism.

### Association of SNPs and response of induction chemotherapy

Among the enrolled 284 AML patients, 191 received a anthracycline-based induction chemotherapy, 108 achieved CR and 83 not CR after the initial induction chemotherapy. We analyzed the correlation between KLK2 SNPs and CR after induction chemotherapy. The chi-square test or Fisher’s exact test was used to evaluate the association between KLK2 rs198977 and response after induction chemotherapy in AML. However, there were no significant differences in genotype and allele frequencies between the CR and non-CR groups (*p* > 0.05, Table 6). Moreover, after analyzing the correlation between KLK2 rs2664155 and the response to induction chemotherapy in AML, there were no significant differences in genotype and allele frequencies between two groups (*p* > 0.05, Table 6).

**TABLE 6:** Association between KLK2 gene polymorphism and CR of induction chemotherapy in AML.

Gene, SNP	Genotype, allele	CR		No CR		Model	χ^2^ test *p*-value
		Count	%	Count	%		
KLK2, rs198977	CC	70	64.81	46	55.42	Co-dominant	0.410
	TC	32	29.63	30	36.14	Dominant	0.188
	TT	6	5.56	7	8.43	Recessive	0.434
	C	172	79.63	122	73.49	Allele	0.158
	T	44	20.37	44	26.51		
rs2664155	GG	96	88.89	73	87.95	Co-dominant	0.709
	AG	12	11.11	9	10.84	Dominant	0.841
	AA	0	0	1	1.2	Recessive	0.435
	G	204	94.44	155	93.37	Allele	0.663
	A	12	5.56	11	6.63		

AML, acute myeloid leukemia; CR, complete response; SNP, single nucleotide polymorphism.

### Association of SNPs and overall survival in AML

**FIGURE 1:**
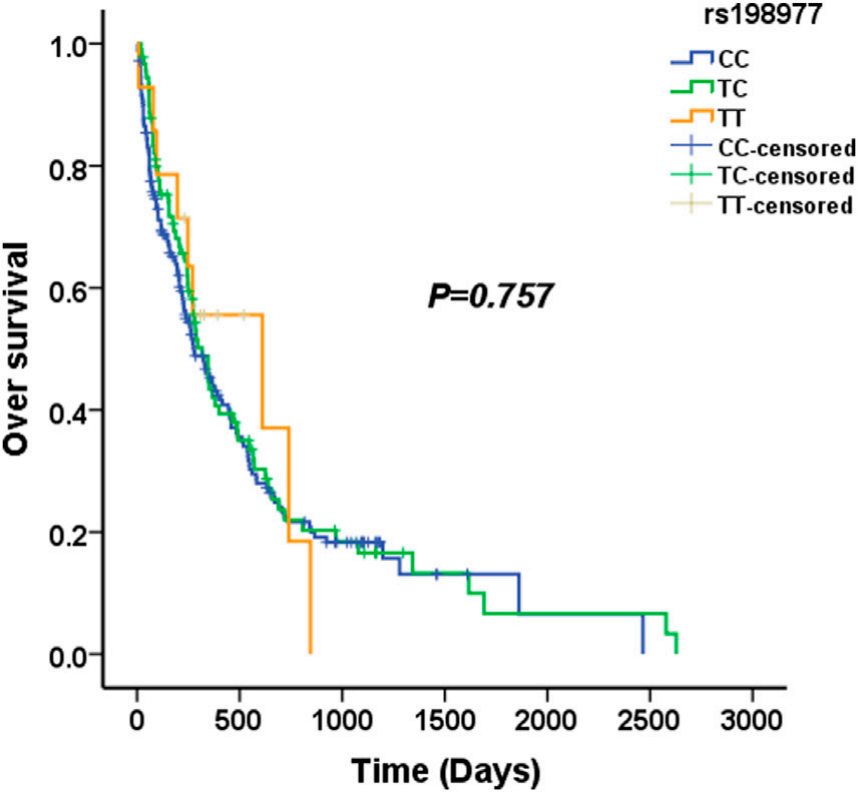
The median overall survival of AML patients with the CC, TC and TT genotypes in rs198977.

**FIGURE 2:**
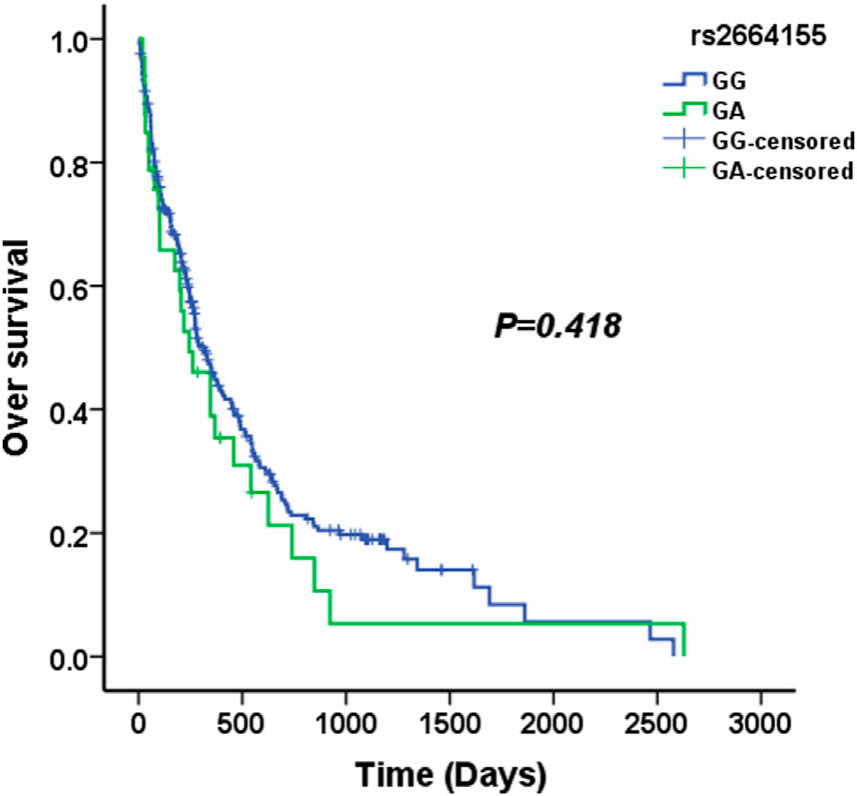
The median overall survival of AML patients with the GG and GA genotypes in rs2664155.

We used four genetic models to analyze the association between KLK2 SNPs and survival in AML patients. The median survival time of KLK2 rs198977 patients was 277 days for CC genotype, 317 days for TC genotype, and 610 days for TT genotype. Kaplan-Meier analysis showed no significant difference in over survival among rs198977 genotypes (*p* = 0.757) (Figure 1). The median survival time of GG genotype of KLK2 rs2664155 patients was 317 days, the median survival time of GA genotype was 244 days, and only 2 patients with AA genotype were not analyzed. Kaplan-Meier analysis showed no significant difference in over survival between GG and GA genotypes in rs198977 (*p* = 0.418) (Figure 2).

## Discussion

In this study, 284 AML patients (non-M3) and 280 healthy controls were recruited from Han Chinese population to investigate the relationship between two SNPS of KLK2 (rs198977 and rs2664155) and the pathogenesis, treatment, and prognosis of AML. Interestingly, our results revealed that KLK2 rs198977 polymorphism was obviously related to AML susceptibility under the TT genetic models. Moreover, AML patients with rs198977 genotype TT have an increased risk of developing hyperleukemia. However, the results of this study showed that KLK2 SNPs (rs198977 and rs2664155) were not associated with risk stratification, response to induction chemotherapy, and survival of AML. Our results suggest that KLK2 may be involved in the pathogenesis of AML and its complication hyperleukocytosis.

KLK2 is one of the members of the human kallikreins family, which consists of 15 family members (KLK1 - KLK15) (Mahabeer and Bhoola, 2000). KLK2 is expressed in many tissues, such as breast, testis, ovary, thyroid and parotid glands, especially in the prostate, which is higher than other tissues (Mahabeer and Bhoola, 2000; Diamandis et al., 2004). Currently, a large number of studies have confirmed that KLK2 is closely related to prostate cancer, including diagnosis, pathological grade, stage and patient prognosis (Bonk et al., 2020; Boyukozer et al., 2020). KLK2 is involved in multiple proteolytic cascades that influence tumor growth and metastasis. Moreover, KLK2 has been found to enhance the proliferation of prostate cancer castration-resistant cells through protease-activated receptors (PAR) (Shang et al., 2014). PAR is G-protein-coupled receptor (GPCR) and KLK2 is a potentially important activator of this receptor and cancer-related mediators (Shang et al., 2014; van Senten et al., 2020). And PAR has been shown to play an important role in the occurrence and metastasis of many cancers, including breast and colon (Bar-Shavit et al., 2016; Covic and Kuliopulos, 2018). PAR can activate the proliferation, invasion and release of angiogenic factors of cancer cells (Bar-Shavit et al., 2016; Covic and Kuliopulos, 2018). Furthermore, many studies have found that abnormal GPCR expression and GPCR-mediated signaling pathway disorders are involved in the development of AML (Selheim et al., 2019). In our study, TT of the rs198977 recessive genotype was significantly related to AML susceptibility. And, it suggested that TT in the recessive genotype of rs198977 played an important role in the pathogenesis of AML. However, the pathogenesis of AML may be related to KLK2, and the mechanism may also be caused by abnormal GPCR expression and GPCR-mediated signaling pathway disorders. But its specific mechanism still needs to be confirmed by further research.

AML patients with WBC count greater than 100 × 10^9^/L are called hyperleukocytosis of AML or hyperleukocytic AML (HAML). AML patients with hyperleukocytosis account for about 18% of AML. AML patients with hyperleukocytosis have a serious condition and a poor prognosis, and often die of various serious complications, such as leukostasis, tumor lysis syndrome (TLS) and disseminated intravascular coagulation (DIC) (Short et al., 2018; Bewersdorf and Zeidan, 2020). Although it is found that there are many risk factors for hyperleukocytosis in AML, even including cytogenetic and molecular biological characteristics, such as M4 and M5, MLL gene abnormalities, and FLT3-ITD mutations (Schoch et al., 2003; Pastore et al., 2015; Shallis et al., 2020). However, the mechanism of hyperleukocytosis in AML, which leads to the crazy proliferation of leukemic cells remains unclear. It is of great significance to explore the pathogenesis and effective treatment of hyperleukocytosis in AML to reduce the early mortality caused by hyperleukocytosis.

In this study, we analyzed the relationship between two important SNPs of KLK2 (rs198977 and rs2664155) and blood count in AML patients. The TT genotype of KLK2 rs198977 was associated with an increased incidence of hyperleukocytosis in AML. Therefore, KLK2 gene may be related to the occurrence of hyperleukocytosis in AML. The specific mechanism leading to the proliferation of leukemia cells remains unclear. However, the hyperleukocytosis of AML must be related to the extreme proliferation and blocked apoptosis of leukemia cells. It was found that KLK2 combined with androgen receptor cofactor (ARA)70 plays an important role in promoting tumor cell proliferation and inhibiting apoptosis (Shang et al., 2014). The combination of KLK2 and ARA70 can reduce G1 arrest of tumor cells and promote tumor proliferation by regulating p21/cdk2/cyclin D1 signaling pathway (Shang et al., 2014). And combination of KLK2 and ARA70 can also inhibit tumor cell apoptosis and promote tumor progression by bax/bcl2/caspase-3 signaling pathway (Shang et al., 2014). The mechanism mentioned above may be the reason for the rampant proliferation of AML cells and the occurrence of hyperleukocytosis, but further studies are needed to confirm it.

Overall, this study identifies a new risk SNP that may serve as a risk biomarker for AML and hyperleukaemia, and it may contribute to new personalized targeted drug therapy regimens for AML. However, it is important to validate these results in a larger sample size of AML and healthy controls.

## Conclusion

In this study we found that the TT genotype of KLK2 SNP rs198977 was associated with increased risk of AML susceptibility. Moreover, the TT genotype of KLK2 SNP rs198977 was associated with a higher incidence of AML hyperleukocytosis. For the first time, our results show that the genetic variants of KLK2 are associated with susceptibility and hyperleukocytosis in AML. This study provides a new perspective on the pathogenesis of AML and hyperleukocytosis, and provides a new direction for exploring its targeted therapy. Due to the heterogeneity of AML patients and the limited number of patients in this study, the results need to be further confirmed in larger studies.

## Data Availability

The original contributions presented in the study are publicly available. This data can be found here: https://www.ncbi.nlm.nih.gov/clinvar/ Accession number: SCV004012899.
